# Cover crops support ecological intensification of arable cropping systems

**DOI:** 10.1038/srep41911

**Published:** 2017-02-03

**Authors:** Raphaël A. Wittwer, Brigitte Dorn, Werner Jossi, Marcel G. A. van der Heijden

**Affiliations:** 1 Plant-Soil-Interactions group, Agroscope, Reckenholzstrasse 191, CH-8046 Zurich, Switzerland; 2 Department of Environmental Systems Science, ETH Zurich, Universitätstrasse 2, CH-8092 Zurich, Switzerland

## Abstract

A major challenge for agriculture is to enhance productivity with minimum impact on the environment. Several studies indicate that cover crops could replace anthropogenic inputs and enhance crop productivity. However, so far, it is unclear if cover crop effects vary between different cropping systems, and direct comparisons among major arable production systems are rare. Here we compared the short-term effects of various cover crops on crop yield, nitrogen uptake, and weed infestation in four arable production systems (conventional cropping with intensive tillage and no-tillage; organic cropping with intensive tillage and reduced tillage). We hypothesized that cover cropping effects increase with decreasing management intensity. Our study demonstrated that cover crop effects on crop yield were highest in the organic system with reduced tillage (+24%), intermediate in the organic system with tillage (+13%) and in the conventional system with no tillage (+8%) and lowest in the conventional system with tillage (+2%). Our results indicate that cover crops are essential to maintaining a certain yield level when soil tillage intensity is reduced (e.g. under conservation agriculture), or when production is converted to organic agriculture. Thus, the inclusion of cover crops provides additional opportunities to increase the yield of lower intensity production systems and contribute to ecological intensification.

Agriculture is facing one of the biggest challenges of our time, namely to produce enough high quality food while reducing external inputs and minimizing negative environmental impacts. Intensive conventional agriculture can contribute to high crop productivity. However, with its excessive use of pesticides and mineral fertilizers, intensive agriculture has a negative impact on the environment by decreasing biodiversity, causing pollution and eutrophication of water, and degrading soil quality^1^,^2^,^3^.

To mitigate this trend, ecological intensification has been proposed^4^,^5^,^6^,^7^. Ecological intensification is defined as the environmentally friendly replacement of anthropogenic inputs and/or enhancement of crop productivity by including agricultural practices that promote regulating and supporting ecosystem services^4^. In Europe, efforts are particularly dedicated to reduce the environmental impact of intensive agriculture and the use of synthetic, anthropogenic inputs. Various strategies and management practices have been suggested that could be used for ecological intensification in arable systems. These include organic farming^8^,^9^, agricultural practices with reduced or no soil tillage (e.g. conservation agriculture^10^), and the use of cover crops instead of longer bare fallow periods^11^,^12^.

Organic farming is proposed for ecological intensification because it promotes biodiversity and soil fertility and has a reduced environmental impact^8^,^13^,^14^,^15^,^16^,^17^. Conservation agriculture (CA), in turn, contributes to soil protection, sustains soil quality, and results in a better use of natural resources^10^. Despite these clear ecological benefits, organic yields^18^,^19^ and yields under conservation agriculture^20^ are often below yields in conventional systems. This yield gap can reduce the positive environmental footprint of organic farming and CA compared to conventional farming because more land is needed to produce the same amount of food. Moreover, although organic and no-tillage agriculture have received increased attention in Europe, and are sometimes actively supported by governmental direct payments (e.g. in Switzerland by FOAG), less than 10% of arable land is actually under no-till or organic agriculture.

Over the last decade, substantial effort has been devoted to implementing CA practices (minimal tillage, permanent soil cover and diverse crop rotation) under organic production because a combination of both strategies could have synergistic effects and further improve soil quality^21^,^22^,^23^. A recent meta-analysis by Cooper *et al*.^24^ concluded that organic yields are not necessarily lower under reduced tillage but that soil carbon storage is improved. However, the application of reduced or no tillage practices often increases problems related to weed control and crop nutrition^25^,^26^,^27^,^28^. One way to tackle these issues is the inclusion of cover crops in the crop rotation.

Cover crops are implemented between two main crops and are known to provide various ecological services in agro-ecosystems, such as protection against soil erosion, reduction of nutrient losses, improvement of soil and water quality, and to some extent, the reduction of weeds and pests^29^,^30^,^31^. Furthermore, adding nitrogen (N) fixing legume species as a cover crop can improve N nutrition of the succeeding main crop and increase the soil N organic pool^32^. Thus, cover crops can contribute to a more sustainable agriculture and alleviate weed and crop nutrition issues related to organic and conservation agriculture. Despite these advantages, cover crops are generally not widely used by farmers, mainly due to additional costs and labour requirements. Moreover, cover crop effects on productivity, crop nutrition, or weed control are variable and depend on cover crop species, soil type, and climate^32^. To date, most research on cover crops has focused on their effects on water quality and N dynamics^29^,^32^ or on the choice of plant species^31^ and management options^33^,^34^ (e.g. sowing and killing techniques and dates). The impact of legume cover crops on productivity is known to be strongly related to the amount of nitrogen fertilization^35^,^36^,^37^, and nutrient mineralisation dynamics and weed control potential is influenced by the type of cover crop and tillage. Nevertheless, so far, few replicated randomized field experiments have tested cover crop effects in different arable systems simultaneously, and little is known about the relative importance of cover crops in different cropping systems.

To address this question, we set up a long-term arable cropping system experiment, known as the Swiss farming systems and tillage experiment (FAST). In this experiment, we compare the effects of four cover crop treatments (a legume cover crop, a non-legume cover crop, a mixture of several species, and a control treatment without cover crops (bare fallow)), simultaneously in four different arable production systems: conventional and organic arable cropping systems, each with intensive tillage (plough) or with soil conservation tillage treatments (no-tillage and reduced tillage for conventional and organic systems, respectively). These four production systems reflect a management intensity gradient where conventional intensive tillage has the highest intensity and organic reduced tillage has the lowest intensity in terms of external anthropogenic inputs and soil disturbance per unit area (Table 1). In the present study, we assessed the short-term effects of cover crops on wheat and maize yield, crop nutrition, and weed infestation.

We hypothesized that:

- The effects of cover crops increase with reduced management intensity as the services provided by cover crops compensate for diminishing intensity.
- Effects of cover crops are highest in the organic reduced tillage system where all three input factors (pesticides, mineral fertiliser and energy use) are absent or have reduced intensity.
- Different cover crops differ from each other in their impact on crop yield, and nitrogen-fixing cover crops (e.g. legumes) enhance nitrogen availability.
- The implementation of cover crops as an ecological management tool enhances productivity across all production systems.

## Results

### Effect of production system and cover crops on grain yield

**Figure 1:**
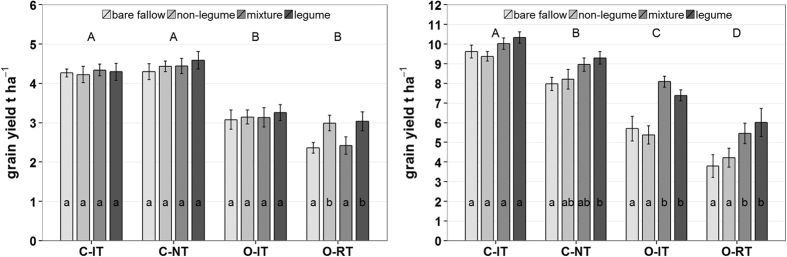
Grain yield affected by production systems and cover crops. Winter wheat (left) and maize (right), (mean ± standard errors, n = 8), (C-IT: Conventional intensive tillage, C-NT: Conventional no tillage, O-IT: Organic intensive tillage, O-RT Organic reduced tillage). Capital letters indicate significant differences among production system and lower case significant differences between cover crop treatments within each production system (Tukey-Test, α = 0.05, for statistical output see Table 2).

Grain yield of wheat and maize varied significantly between the different production systems (Table 2 and Fig. 1). The average yield of maize and wheat was highest in the conventional intensive tillage system (C-IT), intermediate in the no tillage conventional system (C-NT) (−8%) and organic intensive tillage system (O-IT) (−31%), and lowest in the organic system with reduced tillage (O-RT) (−46%). Both crops largely responded the same way to the different production systems, with lowest yields in the organic reduced tillage system and highest yields in the conventional systems. Intensive tillage generally increased yield, both for organic (+23%) and conventional production systems (+8%), especially for maize.

Grain yield in the different production systems also varied depending on the cover crop treatment (Table 2 and Fig. 1). Averaged across all production systems, the use of legume cover crops and cover crop mixtures significantly increased overall yield of wheat and maize by 12% and 11% respectively, compared to the bare fallow treatment. In contrast, no significant effect of the non-legume cover crop (+3% yield increase) could be observed.

**Figure 2:**
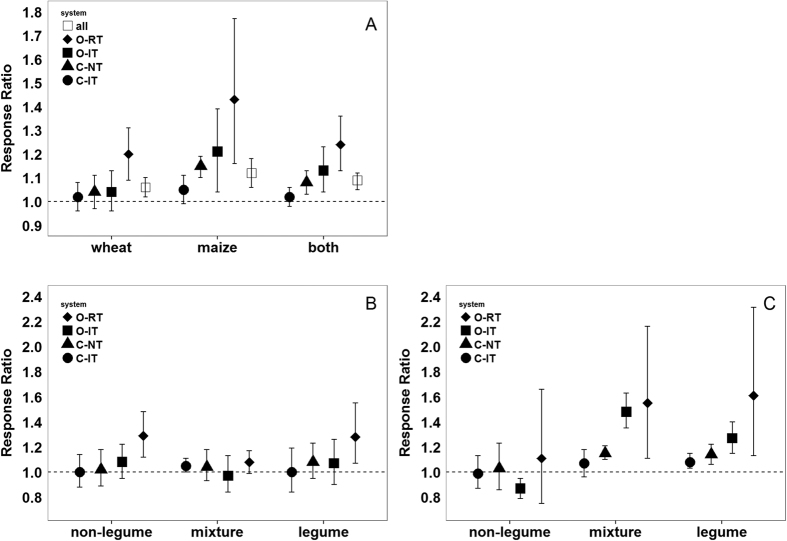
Cover cropping to bare fallow yield response ratio in the different production systems for both crops together (**A**) and for wheat (**B**) and maize (**C**) (C-IT: Conventional intensive tillage, C-NT: Conventional no tillage, O-IT: Organic intensive tillage, O-RT Organic reduced tillage). Mean response ratios and 95% confidence intervals (CI) are shown (n = 8). Means are considered significantly different from bare fallow if their CIs are not overlapping 1.

Significant cover crop effects in wheat were only observed within the O-RT system, where wheat yield was significantly higher after the non-legume (white mustard) and legume (common vetch) cover crop treatments (Figs 1 and 2). The general effect of the legume cover crop on wheat was low but still significantly higher compared to the control treatment across all four production systems (+9%).

Cover crop effects on yield were much higher for maize. Maize yield was increased after the legume cover crop (hairy vetch), compared to bare fallow, in all tested production systems, having 61%, 27%, 14%, and 8% higher yields in O-RT, O-IT, C-NT and C-IT, respectively (Figs 1 and 2). This increase was significant in all systems except C-IT. In contrast, the non-legume cover crop (white mustard) had no significant impact on yield but showed differential effects depending on the cropping systems. Maize yield was not affected by white mustard in the conventional systems. It negatively affected yield in the O-IT system and caused slightly higher yields in the O-RT system. The significant interaction term between production system and cover crop treatment for maize yield (Table 2) further showed that cover crop effects were production system dependent.

Figure 2 shows the mean response ratio of cover cropping compared to bare fallow for each of the four cropping systems. Overall, this ratio tended to increase along the management intensity gradient, being lowest in the most intensively managed system (C-IT) and highest in the most extensively managed system (O-RT). This observation was more pronounced for maize and was mainly driven by the legume and mixture cover crop treatments (Fig. 2).

**Figure 3:**
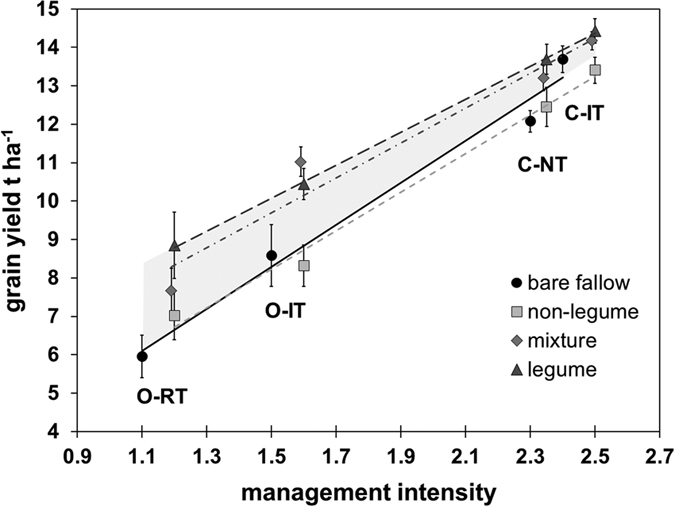
Grain yield (sum of wheat and maize) as a function of management intensity (production system) and cover crop treatments. (Mean ± standard errors, n = 8). The management intensity is derived from Table 1. The grey area shows the potential of cover crops for ecological intensification for each of the four production systems as a function of decreasing management intensity.

The addition of cover crops also altered the yield gap between the four production systems, as illustrated in Fig. 3. For instance, average yield in the conventional production system without tillage was comparable to the intensive tilled system when legume based cover crops (legume and mixture treatments) were present. However, in absence of cover crops, average yield was 10% lower in the system without tillage. Similarly, crop yield in the organic production system with reduced tillage was comparable to the intensively tilled organic system when cover crops (especially legumes) were present. However, in absence of cover crops, crop yield in the organic reduced tillage system was 23% and 33% lower than in the tilled organic systems for wheat and maize, respectively. Overall, cover cropping effects decreased with increasing management intensity.

### Effects of production system and cover crop on weeds

**Figure 4:**
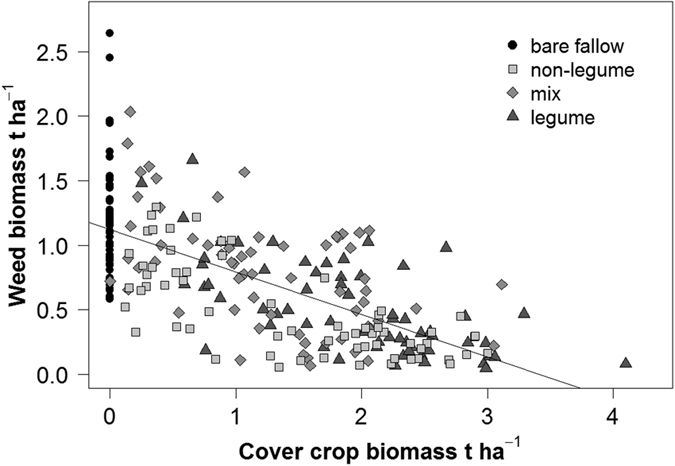
Linear correlation between cover crop biomass and weed biomass. (All data, n = 256, r^2^ = 0.48, p < 0.001).

The addition of cover crops significantly reduced weed biomass during the fallow period for each of the cover crop treatments (Table 2), and cover crop biomass was negatively correlated with weed biomass (Fig. 4). The higher the cover crop biomass, the less weeds could establish, regardless of whether the cover crops were growing short-term (before wheat) or long-term (before maize). Weed reduction reached 50% efficiency with a cover crop biomass production of at least 1.7 t ha^−1^.

Decreased weed biomass during the cover cropping period did not necessarily result in decreased weed pressure in the following main crop. In contrast, cover crop treatments had only a small impact on the weed pressure in the main crops, and weed cover at the critical main crop growth stage depended on the production system (Table 2). While weeds could be successfully controlled with herbicides in the conventional systems (less than 10% weed cover) and relatively well supressed through intensive tillage in the organic system (17% weed cover over both crops), weed cover was much higher in the organic reduced tillage system (23% soil cover in winter wheat and 35% cover in maize) (see Supplementary Fig. S2). However after the white mustard cover crop treatment, which produced the most biomass, weed cover at the critical growth stage was significantly reduced for winter wheat in the O-RT system (F_3,21_ = 6, p < 0.01, see Supplementary Fig. S2). The significant interaction between production system and cover crop treatment on weed cover in wheat (Table 2) also showed that cover crop effects on weeds depended on the production system. In contrast, no differences in weed cover were observed between cover crop treatments in maize for any of the four production systems (see Supplementary Fig. S2).

### Effects of production system and cover crop on crop nutrition

**Figure 5:**
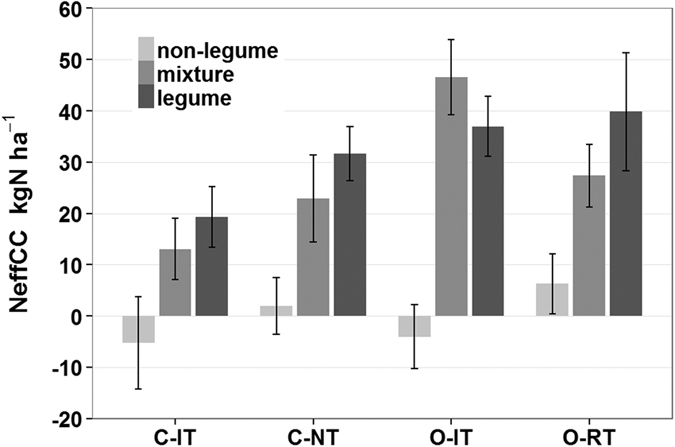
N effect from cover crop on the N uptake of maize (NeffCC) in the different production systems. (mean ± standard errors, n = 8, NeffCC calculation see equation (3)), (C-IT: Conventional intensive tillage, C-NT: Conventional no tillage, O-IT: Organic intensive tillage, O-RT Organic reduced tillage).

Main crop N content was significantly influenced by production system and cover crop treatments (Table 2). Both grain N concentration and N content were higher in the conventional systems and were generally positively affected by the legume cover crop treatment and the cover crop mixture treatment, which also included legumes (see Supplementary Table S3). Maize N uptake was strongly affected by the cover crop treatment, while effects on wheat were negligible (Table 2). In order to assess the contribution of cover crops for crop N uptake, a cover crop N effect (N_effCC_) was computed (see equation (3) in the methods). Averaged across all production systems and compared to bare fallow, the inclusion of the legume cover crop or the cover crop mixture increased maize N uptake (N_effCC_) by 32 kg ha^−1^ and 28 kg ha^−1^, respectively. The non-legume cover crop did not affect N uptake (0 kg ha^−1^), and the N effect was even negative (not significant) in the ploughed systems (C-IT, O-IT). Legume and mixture cover crop effects on maize N uptake were strongest in the organic systems (O-IT: 27.8 kg N ha^−1^, SE: ± 5.4; O-RT: 22.4 kg N ha^−1^, SE: ± 5.4, n = 8) and decreased within the conventional systems, being intermediate in the C-NT (18.2 kg N ha^−1^, SE: ± 4.2, n = 8) system and lowest in the C-IT system (10.8 kg N ha^−1^, SE: ± 3.2, n = 8) (Fig. 5; results for wheat are shown in the Supplementary Fig. S3). N_effCC_ was positively correlated with maize yield in all production systems. However, the amount of variance explained by the N effect of cover crops decreased along the management intensity gradient, being highest in O-RT and lowest in C-IT (O-RT: r^2^ = 0.73***; O-IT: r^2^ = 0.59***; C-NT: r^2^ = 0.53***; C-IT: r^2^ = 0.29**, see Supplementary Fig. S4).

Analysis of ^15^N natural abundance levels in the legume (hairy vetch) and the non-legume (white mustard) cover crop treatments preceding maize indicated that in hairy vetch, 89% of plant N was derived from biological nitrogen fixation. This corresponds to an additional above ground N input of 94 kg N ha^−1^ (see Supplementary Table S4).

**Figure 6:**
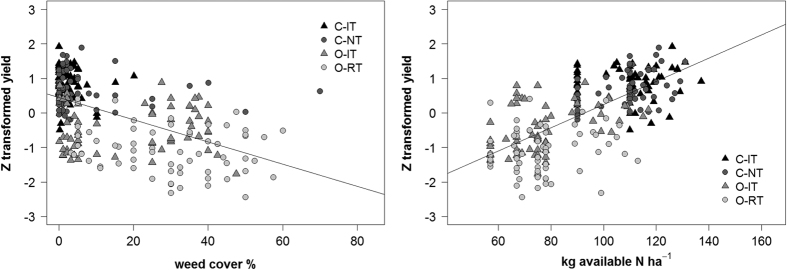
Correlations between the standardized yield of wheat and maize and weed cover (r^2^ = 0.29***) and N supply (r^2^ = 0.48***). Yield values for wheat and maize were standardized across both experiments (FAST I and FAST II) using the z-score to evaluate general effects independently from yield differences among crops and experiments (see materials and methods for N supply estimation). C-IT: Conventional intensive tillage, C-NT: Conventional no tillage, O-IT: Organic intensive tillage, O-RT Organic reduced tillage.

Across all treatments in this experiment, available N supply correlated strongly with yield and explained 48% of the variation in crop yield (Fig. 6). N supply was a function of fertilisation level (supplied available N) and included an estimation of additional N provided by legume cover crops (see methods). The relationship between crop yield and weed cover was weaker and explained only 29% of variance (Fig. 6), indicating that N availability was the main driver of crop yield in the experiment.

### Cover crop growth and biomass production

The legume cover crops, common vetch (before wheat) and hairy vetch (before maize), showed the most stable development across experiments and years (see Supplementary Fig. S5). Both vetch species covered the soil rapidly and reached a high soil cover (on average over 80% soil cover at 60 days after sowing) before wheat and maize. In contrast, white mustard, the non-legume cover crop, showed the highest variation in terms of establishment and growth. White mustard emerged and covered the soil rapidly in the first year of the rotation before wheat (averaged across both experiments over 70% soil cover at 40 days after sowing) but failed before maize in both experiments (less than 20% soil cover at 60 days after sowing). Although emergence was also rapid, further development stopped approximately 25 days after sowing (see Supplementary Fig. S5).

Biomass production differed significantly between cover crop treatments (Table 2). In the first cover cropping period before wheat, white mustard produced the most biomass (2.2 t ha^−1^), followed by common vetch (1.5 t ha^−1^), and the cover crop mixture (0.9 t ha^−1^) (averaged across both experiments). In the second cover cropping period before maize, hairy vetch and the cover crop mixture produced the most biomass (2.4 and 1.6 t ha^−1^, respectively), while white mustard only produced 0.7 t ha^−1^.

## Discussion

Conservation agriculture and organic farming are recognized as valuable strategies to mitigate the negative environmental impacts of arable production. However, in Europe, both systems generally achieve less yields than conventional intensive agriculture, and although adoption of both practices shows a positive trend, arable land under conservation and organic agriculture is, for both systems, less than 5%^38^. The main reasons for this yield gap are difficulties related to weed control and insufficient or asynchronous nutrient availability. Our study confirms this, as yield was highest in the C-IT system and decreased steadily in C-NT, O-IT, and O-RT. The main drivers of the yield decrease were reduced nitrogen availability and increased weed infestation.

Our results demonstrate that cover crops can be used to reduce the yield gap between organic arable farming and conventional farming and between conservation agriculture and intensive tillage. Our observations, thus, confirm the findings of Pittelkow *et al*.^20^, who showed the crucial importance of crop rotation and residue management under no tillage. Moreover, the inclusion of nitrogen fixing cover crops in the organic production systems led to increased yields and could substantially contribute to decrease the yield gaps compared to the conventional systems. In the organic intensively tilled system, yield differences between conventionally managed plots were reduced from −37% without cover crop to −19% with the use of cover crop mixtures. This confirms the statement of Ponisio *et al*.^39^ that diversification practices, such as the use of cover crops to extend the crop rotation, can reduce the yield gap between organic and conventional production. The positive effect of crop rotation diversification with cover crops on maize yield was also shown in a long-term trial in the USA in both organic and integrated management^40^.

A wide range of studies have demonstrated that cover crops provide numerous ecological services including improved crop nutrition, reduced nutrient leaching losses, and enhanced soil and water protection^29^,^30^,^32^,^41^. However, so far it was unclear to what extent the positive effects of cover crops on yield depended on the production system, as few replicated field experiments have directly addressed this question. Earlier studies investigated the effects of cover crops in different crop rotations^11^, with different tillage strategies^25^,^42^, or when fertilizer input varied^35^,^36^,^43^. Our study design enabled us to investigate the magnitude of cover crop effects between highly different production systems. The overall cover crop effect was highest in the O-RT system (+24%), lowest in the C-IT system (+2%), and intermediate in the O-IT (+13%) and C-NT (+8%) systems. Thus, the cover crop effects reflected the management intensity gradient.

Cover crop effects in wheat were only significant in the O-RT system, with the lower management intensity and greater weed reduction likely responsible for the increased wheat yield after the non-legume cover crop. Although it is difficult to separate the various factors that influence yield, it seems that the additional N input by the legume cover crops also compensated for enhanced weed competition in the O-RT system. Indeed, wheat yield after the legume cover crop was equal to that of the non-legume cover crop, despite higher weed cover in this treatment. Moreover, although no differences in weed cover between different cover crop treatments were observed in maize, significantly higher yields were achieved after the legume and mixture cover crops, which support the previous observation.

Biological nitrogen fixation by legume cover crops is likely to be the main mechanism responsible for enhanced maize yield, as both cover crop treatments with legume species (mixture and legume) significantly increased maize yield over the four production systems. This confirms earlier findings that N-demanding crops, such as maize, can greatly profit from additional N input by legume cover crops^32^,^35^,^40^,^43^,^44^,^45^,^46^. The choice of an overwintering legume cover crop also contributed to higher cover crops effects on maize than on wheat, as the growing period of the cover crop before maize was more than twice as high. One of the great advantages of legume cover crops is that their C/N ratio is low so that residues are easily decomposed, thus releasing N rapidly^32^,^41^. Although we did not directly assess the recovery of cover crop or fertilizer N in the main crop, we observed that across all systems approximately 25% of main crop N uptake was derived from the introduction of the legume cover crop. Gentry *et al*.^45^ investigated the impact of a red clover cover crop on corn in conventional, integrated and organic systems and showed that the N credit of red clover on corn was about 40 kg N ha^−1^, which is similar to our results (NeffCC from 19 to 40 kg N ha^−1^). The main difference with our study is that Gentry did not find differences in the magnitude of the N credit between the different management systems they studied. However, no mineral N fertilizer was applied to corn in that study, which could explain why N credit was similar across the systems.

Our results confirm that weed control is a major issue in organic farming, especially under reduced tillage where weed pressure was high. Thus, cover crop management is an important tool in such systems, as it can help to reduce weed pressure, and additional N input by legume species can decrease the competition between weeds and crops for nutrients. This was also observed by Cavigelli *et al*.^44^, who found that N limitation was more important than weed competition when explaining the yield gap between organic and conventional systems. Our results are also in agreement with results from another long-term experiment in Switzerland in which reduced tillage and conventional tillage are compared under organic farming practices. In that experiment, yield in the reduced tillage system was similar or even higher compared to the organic ploughed system. This was due, in part, to the sowing of a legume cover crop (pea) before maize in the reduced tillage system; whereas the cover crop was absent in the ploughed system^47^. However, weed abundance was 2.3 times higher under reduced tillage in that experiment^25^.

The success of cover crops largely depends on proper establishment and biomass production. Several studies showed that weed suppression by cover crops is strongly related to biomass production and early soil cover of the cover crops^31^,^48^. In this study, cover crop success varied greatly across years and experiments, except for the treatment with legume cover crops, which yielded the highest biomass and had lowest yield variability across both years. In contrast, biomass production of the non-legume cover crop was variable, and white mustard did not grow well in the second year of either experiment before maize. Interestingly, more than 80% of the accumulated N in hairy vetch (legume cover crop treatment) was derived from biological nitrogen fixation, suggesting that N availability was low and may explain the reduced growth of white mustard. Moreover, white mustard has a low frost tolerance and was killed during winter, in contrast to hairy vetch, which could continue to grow in the spring.

This study focused on the direct short-term effects of cover cropping on crop yield under a single environment. Whereas cropping system effects on yield are highly influenced by environmental factors^49^, our study enabled us to compare cover crop effects in the different system without this constraint. Thus, we believe that increasing cover crop effects as a function of management intensity should be given greater consideration in the development of cover cropping systems.

In conclusion, our study demonstrates that: i) cover crop effects vary between production systems, ii) that positive effects of cover crops on productivity increase when management intensity is reduced, iii) that cover crop functional groups (legume versus non-legume) have different effects depending on cover cropping length and succeeding main crop, as well as on the production system, and iv) that cover crops are essential to maintain a certain yield level when soil tillage intensity is reduced and/or production converted to organic agriculture. The inclusion of cover crops in the rotation thus provides additional opportunities to increase the yields of production systems with lower management intensity.

## Materials and Methods

### Farming System and Tillage experiment (FAST)

The FAST experiment compares the main arable farming systems in Switzerland, namely conventional and organic farming systems, with different tillage intensity for 6-year crop rotation cycles. In Swiss conventional farming, synthetic fertilizers and pesticides are used for crop nutrition and protection, in contrast to organic farming in which both are prohibited. The conventional systems in FAST are managed according to the “Proof of Ecological Performance” (PEP) guidelines of the Swiss Federal Office for Agriculture. The “PEP” is based on standards for integrated production with requirements for an even nutrient balance, a regular crop rotation, suitable soil protection, and targeted use of plant protection products (FOAG, 2014). Farmers need to follow these guidelines in order to receive direct payments from the government. In 2014, 88% of recorded Swiss farms were registered into “PEP”. The organic systems are managed according to Bio Suisse guidelines, the governing body for organic producers in Switzerland (Bio Suisse, 2016).

The field site is located at the Swiss federal agricultural research station Agroscope, Reckenholz near Zurich (latitude 47°26′N, longitude 8°31′E). The soil type at the experimental site is a calcareous Cambisol and contains on average 1.4% soil organic carbon (SOC), 23% clay, 34% silt, 43% sand, and had a pH(H_2_O) of 7.3. The field site used for this experiment has been cultivated according to Swiss organic standards since 2002, meaning that the organically managed systems experienced no conversion from conventional to organic farming. The long-term (1981–2010) average annual precipitation was 1054 mm, with a mean annual temperature of 9.4 °C (Swissmeteo). FAST is composed of two field experiments established on the same field beside each other (see Supplementary Fig. S1). The first experiment started in summer 2009 (FAST I) and the second in summer 2010 (FAST II), following a staggered start design. Both experiments comprise the following factors: i) production system treatment (conventional intensive tillage (C-IT), conventional no tillage (C-NT), organic intensive tillage (O-IT) and organic reduced tillage (O-RT)) and ii) cover crop treatment (no cover crop as control (C), legume (L), non-legume (NL) and a mixture of several cover crops (M)). This resulted in a total of 16 treatments each replicated four times. The 64 plots for FAST I and FAST II were arranged according to a split-plot design with randomized complete blocks. Each production system (C-IT, C-NT, O-IT, and O-RT) represented a main plot within blocks (see Supplementary Fig. S1). These main plots were each subdivided in four split-plots for the factor cover crop. The size of the main plots is 6 m × 30 m, allowing the use of standard farming equipment. The size of a subplot, which include one cover crop treatment, is 3 m × 15 m. All assessments were performed within the inner 2 m × 10 m of each subplot to avoid border effects.

#### Crop rotation

Before the start of each of the two experiments, the whole experimental area was ploughed and forage pea (*Pisum sativum* L. subsp. *arvense*) was grown as a pre-crop. Subsequently, the experiment started and the following crop sequence implemented using a six-year crop rotation: winter wheat (year 1), maize (year 2), field bean (year 3), winter wheat (year 4), and a grass-clover mixture (year 5 and 6). In the first year of the experiment, cover crops were sown as a short intercrop in the middle of August (see Supplementary Table S1) before sowing of winter wheat (*Triticum aestivum* L. cv. ‘Titlis’). After harvesting winter wheat, cover crops were sown again (see Supplementary Table S1) as a long intercrop before the maize crop (*Zea mays* L. cv. ‘Padrino’). The cover crop treatments consisted of a non-legume (NL) (white mustard, *Sinapis alba*), a legume (L) (common vetch (*Vicia sativa*) before winter wheat and hairy vetch (*Vicia villosa*) before maize), and a cover crop mixture (M) (the mixture UFA-Alpha supplied by UFA-Samen AG containing phacelia (*Phacelia tanacetifolia*), Persian clover (*Trifolium resupinatum*) and berseem clover (*Trifolium alexandrinum*) before winter wheat and a self-designed mixture (SM-ART) containing phacelia, hairy vetch, buckwheat (*Fagopyrum esculentum* Moench) and camelina (*Camelina sativa* L.) before maize). For the main crops, seeds coated with “Coral extra” (Sygenta AG) for wheat and “TMTD 98% Satec” (Bayer AG) for maize were sown in the conventional plots. Untreated seeds were sown in the organic plots, and all seeds were certified. Except for the winter wheat straw, which was removed from the field as is common practice in Switzerland (often used as litter for animal production), all other crop residues (cover crops and maize) remained on the plots. The experiment is ongoing.

#### Soil Tillage and sowing

Primary tillage in the intensive tillage treatment (IT) in both organic and conventional systems was performed with a mouldboard plough (Menzi, B. Schnyder Pflugfabrik, Brütten, Switzerland) to a target depth of 20 cm. This practice is common in Switzerland and most parts of Europe. Subsequently, the seedbed was prepared with a rotary harrow to a depth of 5 cm (Amazone, H. Dreyer GmbH & Co. KG, Hasbergen, Germany) just before sowing. The soil-conservation tillage treatment differed between the conventional and organic systems. In the conventional system, no soil tillage operations were conducted during the whole experimental period, corresponding to no tillage production (NT). Crops were seeded directly into the soil, either with a no-till cereal seeder (Direttissima 250, Gaspardo, Pordeone. Italy) or with a no-till single-grain seeder for maize (Amazone, H. Dreyer GmbH & Co. KG, Hasbergen, Germany). Soil operations in the organic reduced tillage (RT) treatment were performed to a target depth of 5 cm with a disk harrow (Haruwy, Lausanne, Switzerland) before wheat and a rotary harrow before maize. Before sowing of the cover crops, a shallow (5 cm depth) tillage operation was performed with a rotary tiller (Amazone, H. Dreyer GmbH & Co. KG, Hasbergen, Germany), except for the C-NT system in which cover crops were sown directly. Dates of soil tillage operations as well as sowing dates of the crops are given in the supplement (see Supplementary Table S1).

#### Fertilization

Fertilization in the conventional plots was exclusively mineral, and the amount of nitrogen (N) applied was in accordance with the Swiss guidelines for fertilization^50^. Winter wheat and maize received 110 kg N ha^−1^ and 90 kg N ha^−1^, respectively. In addition to N fertilization, phosphorous (P) and potassium (K) were regularly added in the form of P_2_O_5_ and K_2_O, respectively to balance nutrient export by harvested grain and straw (in total 116 kg P ha^−1^ and 138 kg K ha^−1^ for the winter wheat and maize crops, respectively).

The organic plots were fertilized with cattle slurry at a target level of 1.4 livestock units ha^−1^. The slurry was purchased from an organic farmer near the experimental site. Winter wheat received a total of 60 m^3^ ha^−1^ slurry in two applications of 30 m^3^. Likewise, 70 m^3^ ha^−1^ slurry was applied in two applications (40 and 30 m^3^ ha^−1^) in the maize crop. Organic winter wheat received, averaged across both experiments, a total of 119 kg N ha^−1^ (45 kg of slurry N was in the form of directly plant available NH_4_^+^), and maize received 132 kg N ha^−1^ (68 kg of slurry N was in the form of NH_4_^+^). Application dates and the total amounts of applied N are described in the supplement (see Supplementary Table S1). Moreover, we performed an estimation of available N supply by fertiliser and legume cover crops. For the conventional systems N supply from fertiliser (Nfert) corresponded to total synthetic mineral N input. Available N from the slurry (Nfert) in the organic systems is calculated as follows:


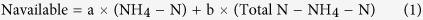


where a (0.8) is the NH_4_-N volatilization coefficient and b (0.35) the proportion of organic N mineralized from the cattle slurry^44^. Finally, an estimation of available N derived from the legume cover crops (NfCC) was determined as follows:


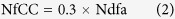


where 0.3 is the proportion of organic N mineralized from the cover crop aboveground biomass and Ndfa the amount of fixed atmospheric N by the cover crops. Ndfa values were determined either from ^15^N analysis for hairy vetch preceding maize (see below) or calculated with values determined by Büchi *et al*.^51^ for the common vetch (legume treatment) and the two clover species preceding wheat. The addition of Nfert and NfCC represent the total N supply.

#### Weed management

Weeds in the conventional plots were managed with post-emergence herbicides, and mechanical control measures were implemented in the organic plots. In the C-NT treatment, Glyphosate (Glyphosat 360 S, Schneiter Agro AG, Switzerland) was additionally applied before sowing of the main crops either to kill natural vegetation (weeds) or the cover crops. The natural vegetation and the cover crops in the other systems were terminated by tillage. For the organic systems, weeds in winter wheat were controlled by a harrow (Lely Holding S.à.r.l., Maasluis, The Netherlands) and by a star cultivator (Haruwy, Lausanne, Switzerland) in maize. Application dates and the number of weed control operations are described in the supplement (see Supplementary Table S1).

### Plant analysis

The grain yield of winter wheat was determined by harvesting the middle 8 rows of each subplot over a length of 10 m with a plot-sized combined harvester (1.33 m width). For maize, the grain yield was determined based on hand collected cobs that were collected in the middle 2 rows of each subplot over a length of 5 m. The overall yields of wheat and maize were relatively low compared to other experiments^52^ and average yields obtained in Switzerland^38^. This can be partly explained by two weather events. In 2010, unsuitable weather conditions delayed the harvest and a storm led to lodging of wheat, which caused, mainly in the conventional plots, at least a 10% yield loss. In July 2011, a hail event led to a wheat yield loss of 15% in organic and 20% in conventional plots in FAST II, as well as an 18–20% maize yield reduction in FAST I (estimates made by hail insurance experts).

Cover crop biomass was determined by collecting the plants within two 0.25 m^2^ frames (50 cm × 50 cm) per subplot before winter onset. For all plant material, dry weight was determined and yield calculated to ton dry weight per hectare. The harvest dates are provided in the Supplementary Table S1. In addition, plant material was ground and the nitrogen concentration of wheat and maize grains determined using elemental analysis by the Dumas method^53^.

In order to assess the contribution of cover crops to N nutrition of the succeeding main crop, a N effect of cover crops (NeffCC) was calculated after Tosti *et al*.^46^:


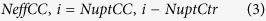


where NuptCC, i is the N uptake of the main crop after a cover crop treatment and NuptCtr is the N uptake of the main crop after the control treatment without cover crops.

Atmospheric nitrogen fixation by legume cover crops was determined with the natural abundance method^54^. The relative abundance of the nitrogen isotope δ^15^N was determined on ground samples of aboveground biomass from the legume and the non-legume cover crop treatments preceding the maize main crop. This was done using mass spectrometry for δ^15^N determination at the stable isotope facilities of the University of Saskatchewan in Canada. The percent N derived from atmosphere (abbreviated as: %Ndfa) was calculated as follows:


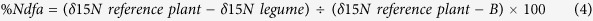


%Ndfa values were calculated using the δ^15^N value of the non-legume cover crop (Brassica) (reference cover crop) growing in the same main plot as the legume cover crop. The B-value for hairy vetch (−0.35) was taken from a study that examined the atmospheric nitrogen fixation of different legume cover crops in Switzerland^51^. The total amount of N fixed from the atmosphere was then obtained by multiplying aboveground N uptake with %Ndfa.

### Weed assessment

Weed cover was assessed for winter wheat at critical growth stage BBCH 25 (tillering) and for maize at critical growth stage BBCH 18 (8 leaves unfolded). The percentage of soil covered by weeds was visually estimated by averaging weed cover in two 1 m^2^ frames located in the middle part of each subplot. Moreover, the growth of the cover crop and their ability to control weeds was assessed using the same protocol and estimating the percentage of soil covered by cover crops and weeds at regular intervals during the cover cropping period. Additionally, cover crop and weed biomass was determined by collecting the biomass within two 0.25 m^2^ frames (50 cm × 50 cm) per subplot. Weed and cover crop species were sorted for each sample before dry weight determination. For all weed assessments, mean values were calculated for each subplot and used for the analysis. An overview of the assessment dates and corresponding crop stages is given in the supplement (see Supplementary Table S1).

### Statistical analysis

Statistical analyses were all performed using R^55^. Variance analyses on the assessed variables were performed using a split-plot design with “production system” (C-IT, C-NT, O-IT, O-RT) as main plot and “cover crop” (C, NL, L, M) as subplot in an ANOVA. The terms “experiment” and “replicate blocks within experiment” were included first in the model to account for their variation. Production system, cover crop treatment and their interactions were considered as fixed effects. The weed cover data were root square transformed prior to analysis to meet analysis assumptions. For graphical visualisation original data are plotted. Significant differences among factor levels were determined by a post-hoc test (Tukey’s HSD test) with the R-package TukeyC^56^ allowing the test to be performed with multiple error terms (main plot error for system and subplot error for cover crop). The Tukey’s HSD test was also used to test for differences among cover crops within each system. In order to assess the effect size of cover cropping on yield among both crops and the four production systems, bare fallow to cover crop mean response ratios were calculated, as well as their 95% confidence interval (CI) values, with the meta-analysis program OpenMEE^57^. Linear regressions were performed to test for effects of weed and N input on yield. Yield data were standardized per experiment and crop by creating a standardized yield (z-transformation) displaying the number of standard deviations of each observation above or below the overall mean using the function “decostand” of the R-package vegan when both crops and/or experiments were analysed together. This made it possible to analyse overall treatment effects irrespective of experiment (year) and crop.

## Additional Information

**How to cite this article:** Wittwer, R. A. *et al*. Cover crops support ecological intensification of arable cropping systems. *Sci. Rep.* **7**, 41911; doi: 10.1038/srep41911 (2017).

**Publisher's note:** Springer Nature remains neutral with regard to jurisdictional claims in published maps and institutional affiliations.

## Supplementary Material

Supplementary Information

## Figures and Tables

**Table 1:** Summary of management practices and management intensity of the four production systems in FAST (C-IT: Conventional intensive tillage, C-NT: Conventional no tillage, O-IT: Organic intensive tillage, O-RT Organic reduced tillage).

Management practices	C-IT		C-NT		O-IT		O-RT	
Tillage	Plough (20 cm), Rotary harrow (5 cm)		—		Plough (20 cm), Rotary harrow (5 cm)		Disk harrow, Rotary harrow (<10cm)	
Weed control	Post-emergence herbicides		Glyphosate, Post-emergence herbicides		Mechanical (Harrow, hoe)			
Fertilization	Mineral (NH_4_-N/NO_3_-N)				Organic (cattle slurry)			
**Management intensity**	**C-IT**		**C-NT**		**O-IT**		**O-RT**	
Cover cropping	**C**	**CC**	**C**	**CC**	**C**	**CC**	**C**	**CC**
Energy use (liter fuel ha^−1^ year^−1^)*	54	61	18	22	47	55	25	32
relative scaling§	**0.9**	**1.0**	**0.3**	**0.4**	**0.8**	**0.9**	**0.4**	**0.5**
N supply (kg N ha^−1^ year^−1^)**	100	100	100	100	69	69	69	69
relative scaling§	**1.0**	**1.0**	**1.0**	**1.0**	**0.7**	**0.7**	**0.7**	**0.7**
Pesticide (kg ha^−1^ active substance)***	2.7	2.7	5.6	5.6	0	0	0	0
relative scaling§	**0.5**	**0.5**	**1.0**	**1.0**	**0.0**	**0.0**	**0.0**	**0.0**
**Averaged intensity score**	**2.4**	**2.5**	**2.3**	**2.4**	**1.5**	**1.6**	**1.1**	**1.2**

Management intensity is estimated for each production system using three anthropogenic input factors (Energy use, weed control, and fertilisation). These factors were also used in different studies evaluating agricultural land use intensity^58,59^. A detailed calculation is included in Supplementary Table S2 online.

^*^Energy use measured as l fuel per ha and year^60^. Includes primary tillage, seedbed preparation, sowing, fertilization, spraying, and mechanical weed control. Sowing (all systems) and mulching (except C-NT) were included as additional management operations for the cover crop treatments.

^**^Supply of plant available N in the organic systems is calculated as in the equation (1). It is assumed that all mineral-N supplied to the conventional system is available to plants.

^***^Pesticide measured as kg applied active substances per ha.

^§^Relative scaling of the input factors among the production systems was calculated relative to the highest value (=1), for the corresponding impact factor.

**Table 2:** Statistical ANOVA output for the assessed variables in winter wheat and maize.

Crop	Parameter	Experiment (E)	Block (E:B)	P. system (PS)	cover crop (CC)	PS × CC
Wheat	Yield	ns	ns	**33.0**_(3,21)_***	**3.6**_(3,84)_*	ns
	Nconcentration	**299.6**_(1,21)_***	**4.0**_(6,21)_**	**37.4**_(3,21)_***	ns	ns
	Nuptake	**26.1**_(1,21)_***	**4.1**_(6,21)_**	**59.4**_(3,21)_***	ns	ns
	NeffCC	ns	ns	ns	**2.4**_**(3,84)**_**°**	ns
	Weed cover in crop	ns	ns	**6.5**_(6,21)_**	**7.4**_(3,84)_***	**4.3**_(9,84)_***
	Cover crop biomass	**154.9**_(6,21)_***	**3.2**_(3,21)_*	ns	**37.9**_(3,56)_***	ns
	Weed biomass in CC	**43.9**_(6,21)_***	**3.1**_(6,21)_*	ns	**45.6**_(3,84)_***	ns
Maize	Yield	**29.3**_(1,21)_***	ns	**75.3**_(3,21)_***	**30.7**_(3,84)_***	**2.7**_(9,84)_**
	Nconcentration	**318.8**_(6,21)_***	**2.2**_**(3,21)**_**°**	**60.9**_(6,21)_***	**53.1**_(3,84)_***	**2.1**_(9,84)_*
	Nuptake	**113.5**_(1,21)_***	ns	**120.0**_(3,21)_***	**43.2**_(3,84)_***	**2.1**_(9,84)_*
	NeffCC	ns	ns	ns	**43.2**_(3,84)_***	**2.0**_(9,84)_*
	Weed cover in crop	ns	ns	**104.6**_(6,21)_***	ns	ns
	Cover crop biomass	**54.4**_(6,21)_***	ns	**3.5**_(6,21)_*	**127.3**_(3,56)_***	ns
	Weed biomass in CC	**35.9**_(6,21)_***	ns	ns	**41.9**_(3,84)_***	ns

(F_(df1,df2)_ values and significance level; df1: numerator degrees of freedom; df2: denominator degrees of freedom; ns: non-significant; °p < 0.1; *p < 0.05; **p < 0.01; ***p < 0.001). NeffCC: N effect of cover crop (see equation (3)). Significant effects of the various factors and treatments are in bold.
