# Supplementary material for: Cover crops support ecological intensification of arable cropping systems
Source: Sci Rep. 2017 Feb 3;7:41911. doi: 10.1038/srep41911 (PMC5291223; doi:10.1038/srep41911)
Supplement: Supplementary Information [file srep41911-s1.pdf]

Supplementary information

Cover crops support ecological intensification of arable cropping systems

Raphaël A. Wittwer[1], Brigitte Dorn[2], Werner Jossi[1], Marcel G.A van der Heijden[1*].

[1] Plant-Soil-Interactions group, Agroscope, Reckenholzstrasse 191, CH-8046 Zurich, Switzerland

[2] Department of Environmental Systems Science, ETH Zurich, Universitätstrasse 2, CH-8092 Zurich, Switzerland

[*]corresponding author: marcel.vanderheijden@agroscope.admin.ch.

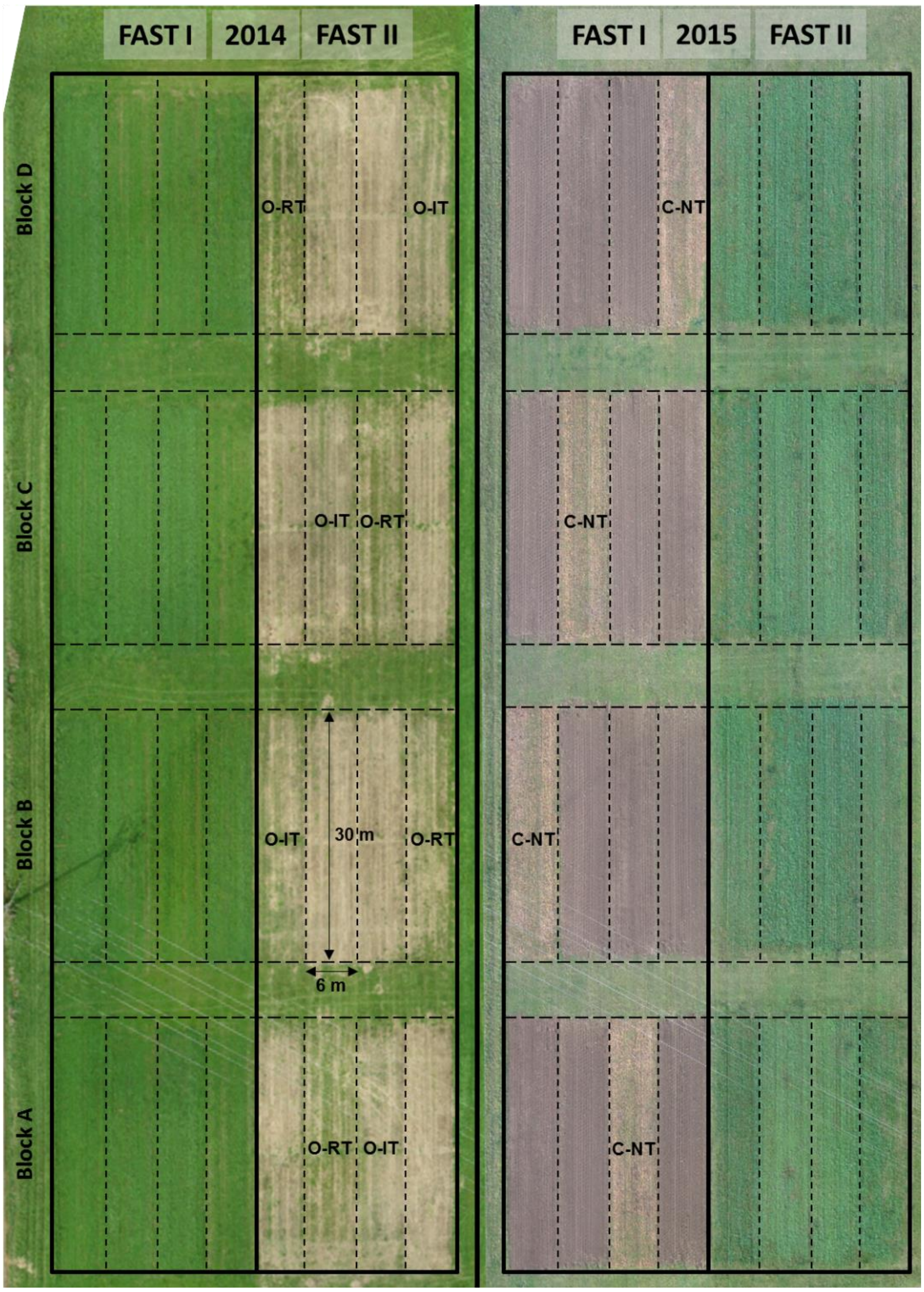

Supplementary Figure S1 I Aerial pictures of the FAST experiment in August 2014 and in October 2015 (orthomosaics computed from drone imagery (eBee AG, senseFly) with the software Postflight Terra 3D (Pix4D)). The picture in 2014 was taken after winter wheat harvest in FAST II. The crop in FAST I is a grass-clover mixture. The picture in 2015 was taken just after winter wheat sowing in FAST I. The crop in FAST II is a grass-clover mixture. Blocks and main plots (dashed lines) are drawn on the picture. The higher weed infestation in the organic reduced tillage system (O-RT) is well visible in FAST II 2014 and the conventional no tilled (C-NT) plots in FAST I 2015 are also noticeable.

Supplementary Table S1 l Field operations, N fertilization and weed assessments. (C-IT: conventional intensive tillage, C-NT: conventional no tillage, O-IT: organic intensive tillage, O-RT: organic reduced tillage).

Trial		FAST I				FAST II			
Crop		Wheat cv. 'Titlis'		Maize cv. 'Padrino'		Wheat cv. 'Titlis'		Maize cv. 'Padrino'	
		product / amount	date	product / amount	date	product / amount	date	product / amount	date
**Cover crop**	non-legume	White mustard		White mustard		White mustard		White mustard	
	legume	common vetch		hairy vetch		common vetch		hairy vetch	
	mixture	UFA-Alpha[1]		SM-ART[2]		UFA-Alpha[1]		SM-ART[2]	
	sowing		24.08.2009		11.08.2010		10.08.2010		11.08.2011
C-IT, O-IT, O-RT	mulching		08.10.2009		15.04.2011		08.10.2010		27.04.2012
C-NT	glyphosate	Glyphosat 360S 3.5 l ha^{-1}	09.10.2009	Glyphosat 360S 4 l ha^{-1}	08.04.2011	Glyphosat 360S 4 l ha^{-1}	08.10.2010	Glyphosat 360S 4 l ha^{-1}	17.04.2012
**Tillage**									
C-IT, O-IT	plough (20cm)		08.10.2009		18.04.2011		09.10.2010		28.04.2012
	rotary harrow (5cm)		20.10.2009		28.04.2011		11.10.2010		04.05.2012
O-RT	disk harrow (5cm)		20.10.2009				12.10.2010		
	rotary harrow (5cm)				29.04.2011				04.05.2012
**Sowing**									
	date	400 seed m^{-2}	21.10.2009	9.5 plant m^{-2}	29.04.2011	400 seed m^{-2}	13.10.2010	9.5 plant m^{-2}	04.05.2012
	row distance	16.6 cm		70 cm		16.6 cm		70 cm	
**Weed control**									
C-IT, C-NT									
	herbicide	Azur 3 l ha^{-1}	25.03.2010	Mikado 1 l ha^{-1}<br>Dasul 1 l ha^{-1}<br>Andil 1 kg ha^{-1}	31.05.2011	Azur 3 l ha^{-1}	16.03.2011	Mikado 1 l ha^{-1}<br>Dasul 1.5 l ha^{-1}<br>Andil 1 kg ha^{-1}	31.05.2012
O-IT, O-RT									
	mechanical 1	harrow	27.04.2010	howing	06.06.2011	harrow	16.03.2011	howing	31.05.2012
	mechanical 2			howing	24.06.2011			howing	20.06.2012
**Harvest (grain)**			04.08.2010		25.10.2011		26.07.2011		22.11.2012
**Fertilization**									
C-IT, C-NT									
	Total N (kg N ha^{-1})	110		90		110		90	
	application 1	60	19.03.2010	30	10.05.2011	60	16.03.2011	30	08.05.2012
	application 2	30	09.04.2010	60	16.07.2011	30	05.04.2011	60	15.06.2012
	application 3	20	17.05.2010			20	09.05.2011		
O-IT, O-RT									
	Slurry N_T / NH_4-N (kg N ha^{-1})*	126 / 50		137 / 67		111 / 40		127 / 68	
	application 1	30 m^{-3} slurry	19.03.2010	30 m^{-3} slurry	12.05.2011	30 m^{-3} slurry	16.03.2011	30 m^{-3} slurry	02.05.2012
	application 2	30 m^{-3} slurry	09.04.2010	40 m^{-3} slurry	16.07.2011	30 m^{-3} slurry	05.04.2011	40 m^{-3} slurry	24.05.2012
**Weed assessments**									
	cover	BBCH 25	24.03.2010	BBCH 18	30.05.2011	BBCH 25	15.03.2010	BBCH 18	01.06.2012
	biomass		07.10.2009		14.10.2010		04.10.2010		18.10.2011

[1] mixture of phacelia (*Phacelia tanacetifolia*), Persian clover (*Trifolium resupinatum*) and berseem clover (*Trifolium alexandrinum*)

[2] self-designed mixture of phacelia, hairy vetch (*Vicia villosa*), buckwheat (*Fagopyrum esculentum* Moench) and camelina (*Camelina sativa* L.)

* N_T: N total

Supplementary Table S2 l Calculations of the management intensity score of the four production systems in FAST (C-IT: Conventional intensive tillage, C-NT: Conventional no tillage, O-IT: Organic intensive tillage, O-RT Organic reduced tillage). Management intensity is estimated for each production system using three anthropogenic input factors (Energy use, N fertilisation and pesticide). These factors were also used in different studies evaluating agricultural land use intensity[1,2].

Production systems<br>Cover cropping	C-IT<br>Control	C-IT<br>CC	C-NT<br>Control	C-NT<br>CC	O-IT<br>Control	O-IT<br>CC	O-RT<br>Control	O-RT<br>CC
**Energy use (liter fuel ha-1 year-1) ***								
sowing (cover crops)		3.8		3.8		3.8		3.8
mulching (cover crops)		3.5				3.5		3.5
primary tillage (plough)	26.1	26.1			26.1	26.1		
seedbed (rotary harrow)	11.5	11.5			11.5	11.5		
shallow tillage (rotary harrow, disc harrow)							14.8	14.8
glyphosate			1.8	1.8				
sowing (main crops)	3.8	3.8	3.8	3.8	3.8	3.8	3.8	3.8
fertilizer applications	10.6	10.6	10.6	10.6				
slurry applications					0.4	0.4	0.4	0.4
herbicide applications	1.8	1.8	1.8	1.8				
mechanical weed control					5.5	5.5	5.5	5.5
total	53.8	61.1	17.9	21.7	47.4	54.7	24.6	31.9
relative scaling §	**0.9**	**1.0**	**0.3**	**0.4**	**0.8**	**0.9**	**0.4**	**0.5**
**N supply (kg N ha^{-1} year^{-1}) ****	100	100	100	100	69	69	69	69
relative scaling §	**1**	**1**	**1**	**1**	**0.7**	**0.7**	**0.7**	**0.7**
**Pesticide (kg ha^{-1} active substance) *****								
Glyphosate			2.88	2.88				
Isoproturon	1.20	1.20	1.20	1.20				
Diflufenican	0.06	0.06	0.06	0.06				
Ioxynil	0.30	0.30	0.30	0.30				
Sulcotrione	0.30	0.30	0.30	0.30				
Terbuthylazine	0.80	0.80	0.80	0.80				
Nicosulfuron	0.04	0.04	0.04	0.04				
total	2.7	2.7	5.6	5.6				
relative scaling §	**0.5**	**0.5**	**1**	**1**	**0**	**0**	**0**	**0**
**Averaged intensity score**	**2.4**	**2.5**	**2.3**	**2.4**	**1.5**	**1.6**	**1.1**	**1.2**

* Energy use measured as l fuel per ha and year[3]. Includes primary tillage, seedbed preparation, sowing, fertilization spraying, and mechanical weed control. Sowing (all systems) and mulching (except C-NT) were included as additional management operations for the cover crop treatments.

** Supply of plant available N in the organic systems is calculated as: a * NH_4-N_{slurry} + b * ($Ntot_{slurry}$ - NH_4-N_{slurry}). a: NH_4-N volatilization coefficient during application (0.8), b: percent of organic N mineralized (0.35)[4]. It is assumed that all mineral-N supplied to the conventional system is available to plants.

*** Pesticide measured as kg applied active substances per ha.

§ Relative scaling of the input factors among the production systems was calculated relative to the highest value (=1), for the corresponding impact factor.

Supplementary Table S3 l Grain yield, N content and N concentration of wheat and maize in different production systems and with different cover crop treatments (mean ± s.e.m.). (C-IT: conventional intensive tillage, C-NT: conventional no tillage, O-IT: organic intensive tillage, O-RT: organic reduced tillage). Different letters indicate significant differences among main factor levels (P. system and cover crop) and among cover crop within each production system (Tukey-Test, α = 0.05). ANOVA output ($F_{(df1,df2)}$ values) is also displayed for each variable (*p<0.05, **p<0.01, ***p<0.001).

P. system	cover crop	grain yield (t ha^{-1})				grain N uptake (kg ha^{-1})			
		Winter wheat		Maize		Winter wheat		Maize	
C-IT		4.3 ± 0.09	a	9.8 ± 0.15	a	107 ± 2.7	a	172 ± 4.3	a
C-NT		4.5 ± 0.09	a	8.6 ± 0.20	b	108 ± 2.5	a	144 ± 5.6	b
O-IT		3.2 ± 0.11	b	6.7 ± 0.29	c	67 ± 4.1	b	102 ± 6.1	c
O-RT		2.7 ± 0.11	b	4.9 ± 0.32	d	56 ± 2.8	b	73 ± 6.2	d
	no	3.5 ± 0.17	a	6.8 ± 0.46	a	82 ± 5.4	a	108 ± 9.1	a
	non-legume	3.7 ± 0.14	ab	6.8 ± 0.43	a	85 ± 4.8	a	108 ± 8.7	a
	legume	3.8 ± 0.16	b	8.3 ± 0.37	b	88 ± 4.8	a	140 ± 8.0	b
	mixture	3.6 ± 0.18	ab	8.1 ± 0.35	b	83 ± 5.6	a	136 ± 7.6	b
C-IT	no	4.3 ± 0.10	a	9.6 ± 0.33	a	105 ± 5.4	a	165 ± 8.7	ab
	non-legume	4.2 ± 0.21	a	9.4 ± 0.23	a	107 ± 5.7	a	160 ± 8.2	b
	legume	4.3 ± 0.23	a	10.3 ± 0.30	a	107 ± 5.8	a	184 ± 7.8	a
	mixture	4.3 ± 0.15	a	10.0 ± 0.30	a	109 ± 5.9	a	178 ± 7.8	ab
C-NT	no	4.3 ± 0.20	a	8.0 ± 0.34	a	106 ± 4.9	a	130 ± 10.7	a
	non-legume	4.4 ± 0.13	a	8.2 ± 0.50	ab	106 ± 5.5	a	132 ± 14.0	a
	legume	4.6 ± 0.22	a	9.3 ± 0.31	b	112 ± 4.4	a	162 ± 7.6	b
	mixture	4.4 ± 0.19	a	9.0 ± 0.33	ab	108 ± 5.3	a	153 ± 9.0	b
O-IT	no	3.1 ± 0.24	a	5.7 ± 0.63	a	67 ± 10.6	a	82 ± 11.4	a
	non-legume	3.2 ± 0.18	a	5.4 ± 0.46	a	68 ± 8.3	a	78 ± 9.8	a
	legume	3.6 ± 0.20	a	7.4 ± 0.28	b	68 ± 6.8	a	119 ± 9.4	b
	mixture	3.1 ± 0.25	a	8.1 ± 0.27	b	66 ± 8.3	a	129 ± 8.4	b
O-RT	no	2.4 ± 0.14	a	3.8 ± 0.59	a	49 ± 3.8	a	55 ± 10.7	a
	non-legume	3.0 ± 0.19	b	4.2 ± 0.49	a	62 ± 5.6	bc	61 ± 9.5	a
	legume	3.0 ± 0.24	b	6.0 ± 0.71	b	65 ± 6.5	c	95 ± 14.7	b
	mixture	2.4 ± 0.22	a	5.5 ± 0.51	b	49 ± 4.9	ab	82 ± 10.3	b
ANOVA									
Experiment (E)		$2.0_{(6,21)}$	ns	$29.3_{(1,21)}$	***	$26.1_{(1,21)}$	***	$113.5_{(1,21)}$	***
Block (E:B)		$1.6_{(6,21)}$	ns	$0.6_{(6,21)}$	ns	$4.1_{(6,21)}$	**	$1.3_{(6,21)}$	ns
P. system (PS)		$33.0_{(3,21)}$	***	$75.3_{(3,21)}$	***	$59.4_{(3,21)}$	***	$120.0_{(3,21)}$	***
cover crop (CC)		$3.6_{(3,84)}$	*	$30.7_{(3,84)}$	***	$2.4_{(3,84)}$	ns	$43.2_{(3,84)}$	***
PS x CC		$1.6_{(9,84)}$	ns	$2.7_{(9,84)}$	**	$1.4_{(9,84)}$	ns	$2.1_{(9,84)}$	*

Supplementary Table S3 l continued.

P. system	cover crop	grain N concentration (g kg^{-1} DM)							
		Winter wheat				Maize			
C-IT		25.1	±	0.6	a	17.4	±	0.2	a
C-NT		24.5	±	0.6	a	16.5	±	0.3	b
O-IT		21.0	±	0.7	b	15.1	±	0.4	c
O-RT		20.8	±	0.6	b	14.5	±	0.4	c
	no	22.9	±	0.7	a	15.3	±	0.4	a
	non-legume	22.8	±	0.7	a	15.3	±	0.4	a
	legume	23.0	±	0.7	a	16.6	±	0.3	b
	mixture	22.7	±	0.8	a	16.3	±	0.3	b
C-IT	no	24.6	±	1.2	a	17.1	±	0.5	ab
	non-legume	25.5	±	1.4	a	17.0	±	0.5	a
	legume	25.0	±	1.2	a	17.8	±	0.4	b
	mixture	25.2	±	1.3	a	17.7	±	0.4	b
C-NT	no	24.8	±	1.3	a	16.1	±	0.7	a
	non-legume	23.9	±	1.5	a	15.7	±	0.8	a
	legume	24.7	±	1.0	a	17.4	±	0.5	b
	mixture	24.6	±	1.4	a	17.0	±	0.5	b
O-IT	no	21.1	±	1.8	a	14.1	±	0.6	a
	non-legume	21.2	±	1.6	a	14.3	±	0.7	a
	legume	21.0	±	1.4	a	16.0	±	0.8	b
	mixture	20.8	±	1.5	a	15.9	±	0.7	b
O-RT	no	21.1	±	1.3	a	13.8	±	0.8	a
	non-legume	20.5	±	1.1	b	14.1	±	0.8	a
	legume	21.2	±	1.2	b	15.4	±	0.7	b
	mixture	20.3	±	1.4	a	14.8	±	0.6	b
ANOVA									
Experiment (E)		$299.6_{(6,21)}$	***			$318.8_{(1,21)}$	***		
Block (E:B)		$4.0_{(6,21)}$	**			$2.2_{(6,21)}$	°		
P. system (PS)		$37.4_{(3,21)}$	***			$60.9_{(3,21)}$	***		
cover crop (CC)		$0.2_{(3,84)}$	ns			$53.1_{(3,84)}$	***		
PS x CC		$0.8_{(9,84)}$	ns			$2.1_{(9,84)}$	*		

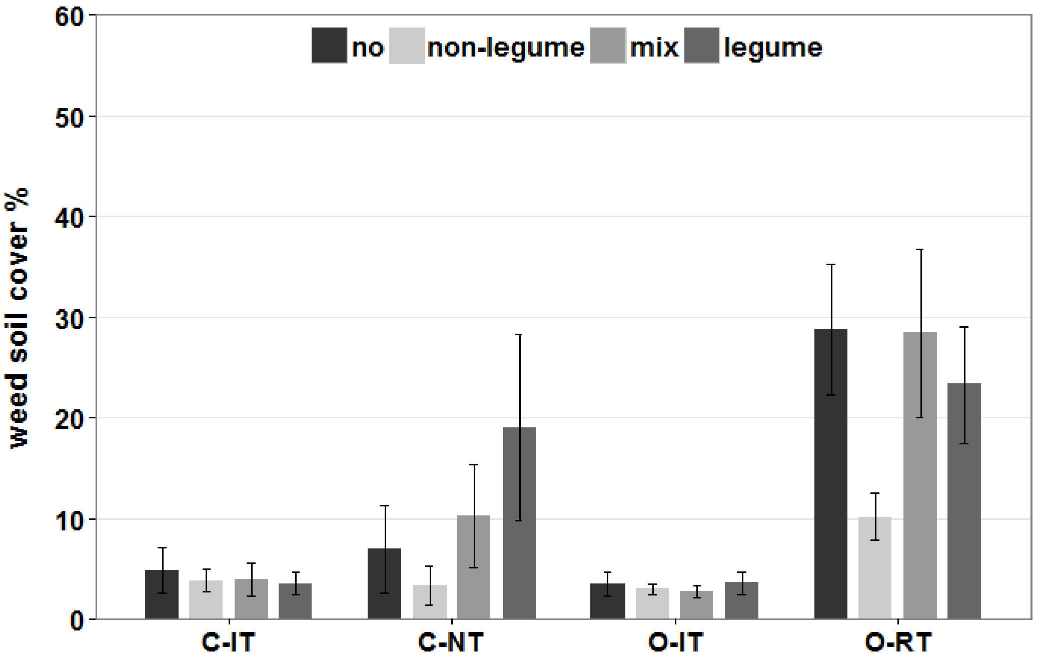

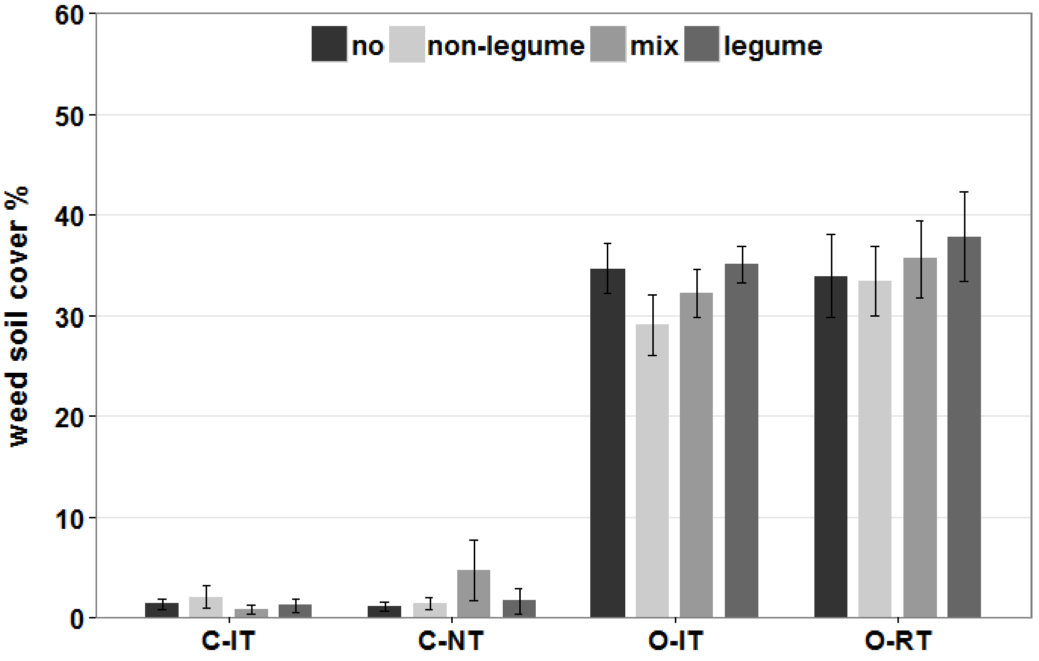

Supplementary Figure S2 l Weed soil cover (%) (mean ± s.e.m., n=8) in wheat at tillering (top) or maize at 8 leaf stage (bottom) for the different production systems (C-PT: conventional intensive tillage, C-NT: conventional no tillage, O-PT: organic intensive tillage, O-RT: organic reduced tillage) in combination with cover crop treatment.

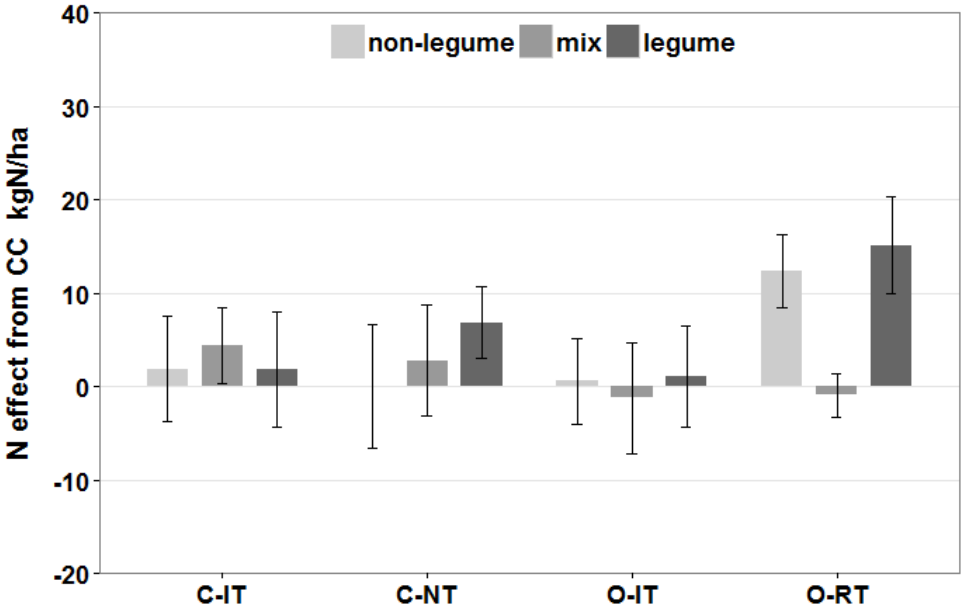

Supplementary Figure S3 l N effect from cover crop on N uptake (NeffCC = Nupt CC - Nupt Control) of wheat (mean ± s.e.m., n=8). (C-IT: conventional intensive tillage, C-NT: conventional no tillage, O-IT: organic intensive tillage, O-RT: organic reduced tillage).

Supplementary Table S4 l Cover crop N content (N total), the fraction of N derived from biological nitrogen fixation (% Ndfa), the amount of N derived from biological N fixation (Ndfa) and the additional amount of N taking up by maize due to the presence of the legume cover crop (NeffCC). Data for the legume cover crop (hairy vetch) before maize are shown (mean ± s.e.m.). (C-IT: conventional intensive tillage, C-NT: conventional no tillage, O-IT: organic intensive tillage, O-RT: organic reduced tillage).

system	Ntot kg/ha				%Ndfa				Ndfa kg/ha				NeffCC [kg/ha]			
C-IT	117	±	13	a	89	±	1.2	a	104	±	11	a	19	±	6	a
C-NT	99	±	9	a	91	±	0.8	a	90	±	8	a	32	±	5	ab
O-IT	111	±	11	a	88	±	1.0	a	98	±	10	a	37	±	6	ab
O-RT	93	±	6	a	89	±	1.5	a	83	±	6	a	40	±	12	b

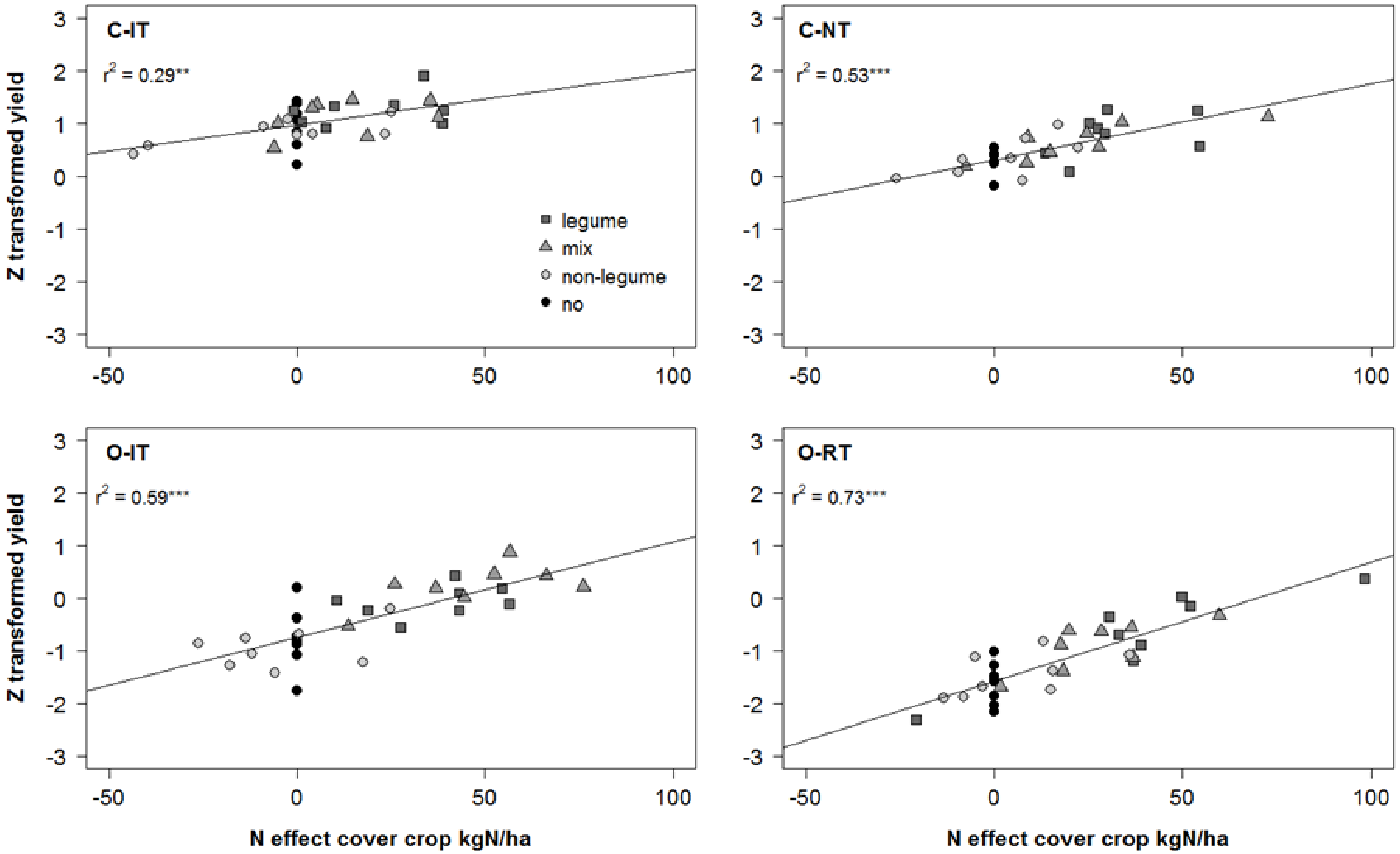

Supplementary Figure S4 | Correlations between N effect from cover crop and the standardized (z-transformed) yield of maize for each of the four productions systems. Yield values for maize were standardized across both experiments (FAST I and FAST II) to evaluate the effects of cover crop independently from yield differences among experiments. The N effect of the cover crop was calculated according to equation (1). R-squared for each system is displayed with individual significance levels (**$p<0.01$, ***$p<0.001$)

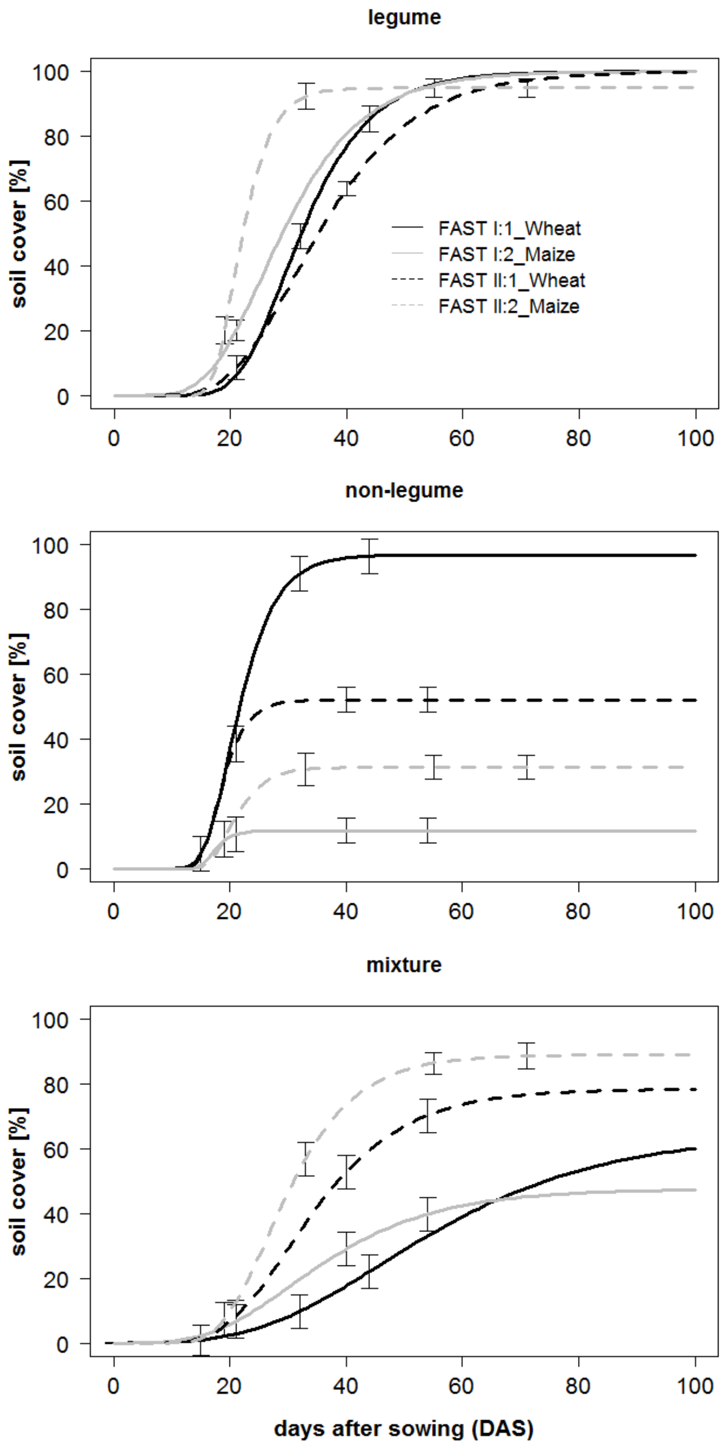

Supplementary Figure S5 l Cover crop growth curves for the legume (common vetch and hairy vetch), non-legume (white mustard) and the cover crop mixtures in each trial (FAST I, FAST II) for wheat and maize. Curves are fitted with the Gompertz growth function of the R-package drc [5]. The model-based standard errors used for the error bars are calculated as the fitted value plus/minus the estimated error, times the 97.5% quantile in the t distribution with degrees of freedom equal to the residual degrees of freedom for the model.

References:

1 Herzog, F. *et al.* Assessing the intensity of temperate European agriculture at the landscape scale. *European Journal of Agronomy* **24**, 165-181 (2006).

2 Ruiz-Martinez, I., Marraccini, E., Debolini, M. & Bonari, E. Indicators of agricultural intensity and intensification: a review of the literature. *Italian Journal of Agronomy* **10**, 74-84 (2015).

3 Nemecek, T. & Kägi, T. Life Cycle Inventories of Swiss and European Agricultural Productions Systems. Final report ecoinvent V2.0 No. 15a. (2007).

4 Cavigelli, M. A., Teasdale, J. R. & Conklin, A. E. Long-term agronomic performance of organic and conventional field crops in the mid-Atlantic region. *Agronomy Journal* **100**, 785-794 (2008).

5 Ritz C. & Streibig, J. C. Bioassay analysis using R. *Journal of Statistical Software* **12** (2005).
